# Mechanism of Notch Signaling Pathway in Malignant Progression of Glioblastoma and Targeted Therapy

**DOI:** 10.3390/biom14040480

**Published:** 2024-04-15

**Authors:** Shenghao Wang, Sikuan Gu, Junfan Chen, Zhiqiang Yuan, Ping Liang, Hongjuan Cui

**Affiliations:** 1 Cancer Center, Medical Research Institute, Southwest University, Chongqing 400716, China; collapsar@email.swu.edu.cn; 2 State Key Laboratory of Resource Insects, Southwest University, Chongqing 400716, China; gsk18753792070@email.swu.edu.cn (S.G.); cjf164964@email.swu.edu.cn (J.C.); yzqyzq1314@email.swu.edu.cn (Z.Y.); 3 Department of Neurosurgery, Children’s Hospital of Chongqing Medical University, Chongqing 400014, China

**Keywords:** glioblastoma, Notch, biological functions, molecular mechanisms, clinical

## Abstract

Glioblastoma multiforme (GBM) is the most aggressive form of glioma and the most common primary tumor of the central nervous system. Despite significant advances in clinical management strategies and diagnostic techniques for GBM in recent years, it remains a fatal disease. The current standard of care includes surgery, radiation, and chemotherapy, but the five-year survival rate for patients is less than 5%. The search for a more precise diagnosis and earlier intervention remains a critical and urgent challenge in clinical practice. The Notch signaling pathway is a critical signaling system that has been extensively studied in the malignant progression of glioblastoma. This highly conserved signaling cascade is central to a variety of biological processes, including growth, proliferation, self-renewal, migration, apoptosis, and metabolism. In GBM, accumulating data suggest that the Notch signaling pathway is hyperactive and contributes to GBM initiation, progression, and treatment resistance. This review summarizes the biological functions and molecular mechanisms of the Notch signaling pathway in GBM, as well as some clinical advances targeting the Notch signaling pathway in cancer and glioblastoma, highlighting its potential as a focus for novel therapeutic strategies.

## 1. Introduction

GBM, which originates from glial cells in the brain, is a malignant tumor known as the most deadly and invasive primary brain tumor [1]. It typically arises in the brain and is a relatively common type of neurological tumor [2]. Treatment of GBM presents significant challenges due to its deep-seated location within the brain tissue, making complete removal difficult [3]. In addition, the highly invasive nature of tumor cells often leads to their spread into surrounding healthy tissues, making surgical intervention difficult [4]. As a result, the 5-year survival rate for patients is only 5%, and the median survival time is 14.6 months, ultimately resulting in a mortality rate of nearly 100% [5]. Current treatment modalities primarily involve curative approaches, including surgical resection, radiotherapy, and chemotherapy, but several emerging adjuvant therapeutic approaches are also on the rise [6]. Different people with GBM have different symptoms [6]. This is because genetic and molecular differences can make treatments less or more effective, making it more difficult to target specific tumor cells [7]. Therefore, there is an urgent need for a deeper understanding and exploration of the mechanisms that drive the malignant progression of GBM. This review has the potential to provide new methods for the therapy of GBM.

The Notch signaling pathway is an evolutionarily conserved cellular signaling mechanism that contributes to diverse biological processes and tissue formation [8]. It governs cell proliferation, apoptosis, tissue homeostasis, survival of stem cells, and cell decisions about cell destiny [9,10,11,12]. The Notch signaling pathway initiates a sequence of signaling occurrences by binding to ligands and activating Notch receptors [13]. GBM development and occurrence have been revealed to be tightly correlated with the Notch signaling pathway [14]. Activation of Notch signaling in GBM promotes cell proliferation and boosts survival capabilities [15]. In certain situations, the initiation of Notch signaling may lead to the differentiation of tumor stem cells, giving rise to various cell types [16], potentially influencing tumor heterogeneity and treatment resistance [17]. Simultaneously, Notch signaling activation may impact surrounding cells, angiogenesis, and immune responses, thereby influencing tumor growth and dissemination [18]. This study aims to present an in-depth review of the molecular mechanisms and biological roles of the Notch signaling system in the malignant progression of GBM in recent years. Although there are numerous Notch inhibitors and other molecular drugs that can effectively slow the growth of GBM by inhibiting the Notch signaling pathway, ongoing clinical trials are still limited. Thus, this review may offer valuable insights into targeted therapeutics and clinical applications of GBM.

## 2. Notch

**Figure 1:**
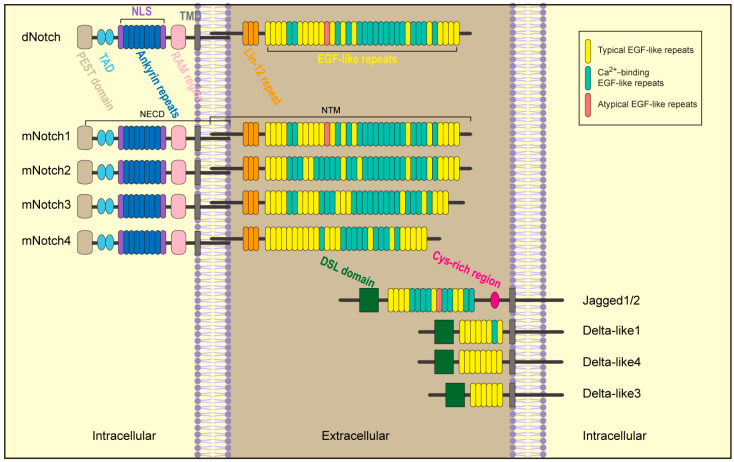
Structure of Notch receptors and ligands.

Notch receptors and their corresponding ligands make up the Notch signaling pathway (Figure 1). Mammals possess four different types of Notch receptors, called Notch1-4. These receptors bind two different families of ligands, the Delta-like ligand (DLL) family and the Jagged (JAG) family. The ligand families consist of DLL1/3/4 Jagged1/2 [19]. Receptors and ligands are transmembrane proteins that can only cross the cell membrane once and interact through extracellular structural domains.

Several repeating epidermal growth factor (EGF)-like structures and the Lin-12/Notch repeats comprise the extracellular domain of the Notch receptor [20,21,22]. This area facilitates the non-covalent bonding between membrane-anchored intracellular Notch and extracellular Notch [23]. The Notch intracellular domain (NICD) comprises a transcriptionally active domain (TAD), an ankyrin repeat domain (ANK) with several repeating ankyrin units, a RBPJK-associated molecule (RAM), and a PEST region that facilitates faster NICD degradation [24]. Unlike the Notch receptor, Delta and Jagged ligands exhibit remarkable similarity: they do not have intracellular domains, and their extracellular portions consist of two parts, 6-16 EGF-like repeats [20] and a distant cysteine Delta/Serrate/Lag-2 (DSL)-rich region [21]. The DSL domain is accountable for interacting with the Notch receptor [25]. Additionally, Jagged1/2 similarly has a region close to the cell membrane that is rich in cysteines [26]. 

**Figure 2:**
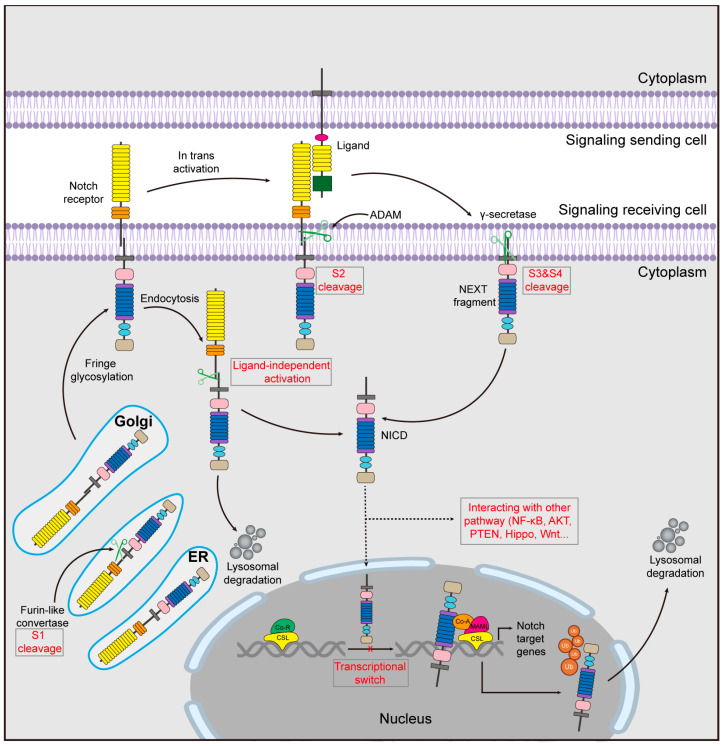
Schematic representation of the Notch signaling pathway. The inactive monopeptide precursor is synthesized in the endoplasmic reticulum and transported to the Golgi where it is cleaved by furin converting enzyme (S1 cleavage) and transported to the cell membrane, where it binds to the ligand, where it is cleaved a second time by a member of the ADAM family (S2) to form the membrane-bound Notch Exhaustive Notch Transcript (NEXT), which is further processed by the precursor-independent γ-secretase complex at two sites (S3 and S4) to generate the active form of the Notch receptor. This fragment is further processed by the precursor-independent γ-secretase complex at two sites (S3 and S4) to generate the Notch intracellular structural domain (NICD), the active form of the Notch receptor, which enters the nucleus and exerts its transcriptional activity in the transcriptional regulation of downstream target genes. Ubiquitylation of the NICD leads to its proteasomal degradation. In addition, mature Notch receptors can return to the cytoplasm via endocytosis, activate introns by interacting with certain proteins lacking the DSL structural domain, and directly participate in other signaling pathways.

In the canonical Notch signaling pathway, the initiation of Notch signaling involves a series of complex mechanisms and processes (Figure 2). Notch receptor protein is produced in the endoplasmic reticulum (ER) and subsequently transported to a type I transmembrane protein in the trans-Golgi apparatus. Here, through cleavage by a furin-like enzyme, it is divided into two fragments [27]. The initial cleavage site (S1) facilitates Notch receptor maturation, forming heterodimers for translocation to the cell membrane [27]. During translocation, the receptor undergoes a conformational alteration, which reveals the second cleavage site (S2) to ADAM10 or ADAM17/TACE metalloproteinases [28]. As a result, the Notch receptor is cleaved into a cytoplasmic extracellular fragment (Notch extracellular structural domain, NECD) and a membrane-anchored fragment (Notch transmembrane structural domain, NTM) [29]. The membrane-anchored Notch truncated (NEXT) fragments are further processed by the γ-secretase transmembrane component at two sites (S3 and S4) [30,31]. The process of cleavage leads to the liberation of the active form of NICD into the cytoplasm. Upon further translocation to the nucleus [32], NICD immediately binds to CSL proteins (CBF1/KBPF2/RBPJK in mammals), transforming the “co-repressor component” into a “co-activator component”, thereby forming a multi-protein-DNA complex. The complex is capable of recruiting nuclear transcriptional coactivators such as Ski-interacting protein (SKIP), mastermind-type (MAML) protein, and the histone acetyltransferases PCAF/GCN5 and CBP/p300 [33,34,35]. This promotes the transcriptional repressor hair enhancement splitter protein (Hes) family and HES-related proteins (Hey), which play a crucial role in determining cell lineage commitment [36,37]. Other Notch target factors include NF-κB, IGF1-R, Cyclin D1 and D3, p21/Waf1, c-Myc, HER2, Notch-regulated amygdaloid repeat-sequence protein (NRAR), pre-Tα, SLUG, survivin, SOX2, and PAX5 [38,39,40,41,42]. The Notch-mediated transcriptional activation process culminates in the degradation of the NICD [43]. This degradation mechanism entails the phosphorylation of a designated region known as the degron, situated within the PEST region of NICD. Cyclin-dependent kinase 8 (CDK8) facilitates this phosphorylation, and subsequently, E3 ubiquitin ligases SEL10 (or FBW7) target the phosphorylated NICD for degradation through the proteasome pathway [44].

Another form of Notch signaling activation occurs in the non-canonical Notch signaling pathway. Mature Notch receptors can be returned to the cell membrane by endocytosis, degraded in lysosomes, or activated in introns through interaction with certain proteins lacking the DSL domain [45], which include membrane-integral proteins such as membrane-linked glycosylphosphatidylinositol (GPI) proteins like NB3/Contactin6 [46], Delta/Notch-like EGF Repeat-containing Receptor (DNER) [47], and secreted proteins such as MAGP1 and MAGP2 [48]. This mode of activation can upregulate immune-related genes and significantly impact T cell development [49]. Furthermore, NICD has the ability to directly engage with the NF-κB, PTEN, PI3/AKT, Hippo, Wnt, or TGF-β signaling pathways both in the cytoplasmic and nuclear domains. This enables the regulation of target gene transcription without the necessity of CSL involvement [50].

Moreover, upon S3 cleavage and the subsequent release of NICD, membrane-bound Notch has the potential to activate the PI3K-AKT pathway [51]. This activation, in turn, promotes the transcription of *Interleukin-10* and *Interleukin-12* [52]. NOTCH3 can induce apoptosis in tumor endothelium cells, regardless of the processes of cleavage and transcriptional regulation [53]. In the absence of binding to Notch receptors, the intracellular region of JAG1 displays the ability to promote epithelial–mesenchymal transition (EMT) and tumor growth [54]. The non-canonical pathways of the Notch signaling cascade offer distinct biological functions, highlighting the diversity within this intricate signaling network.

## 3. Notch Is Involved in Development and Progression of Glioblastoma

### 3.1. Proliferation and Growth

Tumor cells have the ability to replicate indefinitely [55]. Notch signaling is essential to regulating the normal growth and proliferation of many tissues and cell types [56]. In human cancers, one of the most characteristic oncogenic functions of the Notch pathway is its ability to activate the transcription of genes that support cell proliferation. For example, Notch can enhance the expression of Myc, a global regulatory factor that promotes growth metabolism [57,58]. Additionally, Notch signaling has been proven to interact with many other pro-growth pathways with oncogenic activity [59], such as NOTCH1 regulates PTEN expression and PI3K-AKT signaling pathway activity in normal thymocytes through transcriptional networks [60]. 

Research has shown that both Notch1 and its ligands are highly expressed in numerous cell lines of glioma and primary human glioma [61,62]. Targeting Notch1 and its ligands with siRNA can impair the proliferation and growth capabilities of GBM cells [63]. Furthermore, the proliferative capacity of glioma stem cells can be reduced by inhibiting Notch signaling through the use of γ-secretase inhibitors, which act as blockers of this pathway [64,65]. 

Some genes have been confirmed to impact the growth and proliferation of GBM by affecting the Notch pathway. For instance, CRMP5 promotes GBM growth by blocking the lysosome-dependent degradation pathway of Notch pathway-related receptors [66]. RND3, an endogenous inhibitor of the Notch transcriptional complex, can enhance the signaling activity of the Notch pathway through its downregulation, thereby promoting the proliferation of glioblastoma cells [67]. *MiR-34a* can inhibit GBM growth by targeting and inhibiting *Notch1* and *Notch2* gene expression [68]. Leptin promotes glioblastoma cell growth by activating its downstream effectors and target molecules through upregulation of the Notch 1 receptor [69]. NICD is upregulated in human GBM, and it promotes glioblastoma cell proliferation through the Notch signaling pathway [70]. *LINC01152* can recruit SRSF1 and sponge *miR-466* to activate MAML2, thereby promoting GBM progression through the Notch signaling pathway [71]. DLL4 expression in GBM activates host stroma/endothelial Notch signaling, improves intratumoral vascular function, and promotes tumor growth [72]. ZSP inhibits GBM growth in vitro and reduces the stem cell markers Nestin and CD133 by downregulating the Notch2 receptor, which targets Hairy/Enhancer of Split 1 (Hes1) to induce apoptosis. [73]. *MiR-148a* and *miR-31* can target a common factor of Hypoxia-Inducible Factor 1 Inhibitor α through Notch signaling to promote GBM growth [74].

Chemical substances and hypoxic conditions can also affect the proliferation and growth of GBM through the Notch pathway. For example, arsenic trioxide (ATO) can decrease the phosphorylation and activation of STAT3 and AKT through the Notch pathway, thereby reducing the proliferation of glioblastoma cells [75]. Hypoxic conditions can induce the expression of TRPC6 via the Notch pathway. The increase in intracellular calcium ions mediated by TRPC6 leads to enhanced NFAT activation and increased proliferation of GBM cells [76]. 

In summary, there is consistent evidence that aberrant Notch signaling promotes GBM cell proliferation and expansion by regulating downstream effectors or other pathways.

### 3.2. Migration and Invasion

Aberrant Notch signaling plays an important role in the migration and invasion of GBM. Notch1 may affect the invasiveness of GBM through the action of the downstream target gene *Hes1* [77]. Hey1, a downstream effector of the Notch pathway, enhances the invasive and migratory capacities of glioblastoma by suppressing the transcription of the deubiquitinase USP11, which is associated with promyelocytic leukaemia (PML) [78,79,80]. Notch2 transactivates *TNC* genes and promotes GBM invasion in an RBPJK-dependent manner [81]. Combining Notch inhibitors with resveratrol to target CDK4 can modulate the invasiveness of glioblastoma [82,83]. The downregulation of CBF1, a primary transcriptional regulator [84], can reduce Notch signaling activity and thereby attenuate GBM invasion [85].

Specific genes can impact the ability of GBM to migrate and invade by influencing the activity of the Notch pathway. Downregulation of uPA/uPAR can inhibit cleavage of the Notch receptor between the Gly1743 and Val1744 sites, thereby suppressing Notch pathway activity. This inhibition also leads to the downregulation of NF-κB, ERK, and AKT pathways induced by Notch signaling, further inhibiting the migration and invasion of glioblastoma [86]. RBM8A can enhance the invasive capabilities of glioblastoma by stimulating the transcriptional activation of Notch1 and STAT3, thereby activating the Notch/STAT signaling pathway [87,88]. EIF44A3 can promote GBM growth and invasion by regulating Notch1 expression through the STAT3-related pathway [89].

Notch signaling can also affect the invasion and migration capabilities of GBM by influencing other signaling pathways. Notch1 can activate the AKT pathway, thereby enhancing the signaling of β-catenin and NF-κB to promote glioma cell migration and invasion [90,91], and the CXCL1/CXCR4 system can also enhance the migration and invasion capacity of GICs [92]. RBPJ, a key transcription factor in the Notch pathway [93], can enhance the migration and invasion capacity of glioblastoma cells. It does this by augmenting the IL-6-STAT3 pathway and activating primary mesenchymal transformation (PMT) [94]. Additionally, Notch signaling can promote EMT, thereby enhancing the invasiveness and migratory capabilities of tumor cells [95,96]. Activation of the Notch signaling pathway can enhance the expression of Snail and Slug [97].

### 3.3. Apoptosis

The high growth potential and reduced susceptibility to apoptosis in GBM cells are mainly attributed to gene amplification or mutation of oncogenes or pro-apoptotic genes [98]. DLL3 regulates Notch signaling in a negative or modulatory manner within the cell [99]. Downregulation of fibulin-3 mediates an antagonistic interaction with DLL3, leading to diminished Notch signaling and increased apoptosis in glioblastoma cells [100]. *LINC01152* regulates GBM cell apoptosis by positively modulating RBPJ/MAML2 through sponging *miR-466* and recruiting SRSF1 [71]. Overexpression of Notch-1 and EGFR induces apoptosis in glioblastoma cells [101]. 

Some studies suggest that microRNAs can regulate Notch signaling to induce apoptosis in glioblastoma cells. For example, *miR-326*, which is ectopically expressed in glioma stem cells and gliomas, targets the Notch signaling pathway, induces apoptosis, and reduces its metabolic activity [102]. The overexpression of *miR-34c-3p* leads to the upregulation of Notch pathway members, causes S-phase arrest, shortens the G0/G1 phase and induces apoptosis in U251 and U87 cells [103]. *MiR-145* targets BNIP3, and Notch signaling to induce apoptosis in glioma cells [104].

In addition, inhibitors of Notch signaling and some other drugs can also induce apoptosis in glioblastoma cells. For example, DAPT completely blocked trans-4-[4-(3-adamantan-1-yl-ureido)-cyclohexyloxy]-benzoic acid (t-AUCB)-induced phosphorylation of p38 MAPK, MAPKAPK2, and Hsp27 in glioblastoma cells [105]. U-87 MG cells express Fas receptors on their cell membrane, and γ-secretase inhibitors can reduce Fas activation-induced apoptosis mediated by p75(NTR)–Fas receptor interactions [106]. ATO simultaneously inhibits Notch and Hedgehog target genes and promotes apoptosis [107]. Bacoside A leads to a 0.05-fold decrease in *Notch1* gene expression and a 25-fold increase in *Hes1* gene expression in U-87 MG cells, inducing cell cycle arrest and apoptosis [108]. Hypocretin-1 treatment effectively reduces the expression of NCID in glioblastoma cells and induces apoptosis [109]. RSV leads to the expression of low-activity Notch-1 and heterozygous p53 mutations in A172 and T98G cells, inducing apoptosis [110].

### 3.4. Regulation of Stemness

Cell stemness refers to a group of cells that are characterized by their ability to self-renew and differentiate into multiple lineages [111]. Upon activation, Notch receptors release the activated NICD to regulate the expression of Hes and Hey family genes, thereby affecting cell differentiation and maintaining cell stemness [112]. Dysregulation of the Notch pathway leading to abnormal activation of GSCs contributes to malignant glioblastoma formation.

The exogenous expression of CDK4 mutants negates the inhibitory effect of RSV and RO4929097 on the motility/invasion of glioblastoma cells and enhances the expression of stemness-specific markers GFAP, CD13.3, and SOX2, and the EMT markers TWIST and SNAIL, as well as the size/formation capability of the neurosphere [83]. The deletion of the α-kinase structural domain in TRPM7 (M7-DK) and the K1648R point mutation (M7-KR) affect Notch activity and the expression of CD133 and ALDH1 in GSCs [113]. Multiple genes in GBM cells affected by nearby endothelial cells (EC) are dependent on Notch-mediated signaling to maintain GSC in an undifferentiated state [114]. Infection with Human Cytomegalovirus (HCMV) induces the malignant transformation of tumor cells, sustaining stemness by upregulating Notch1 and NICD expression in U251 cells [115]. ATO-mediated Hh/Notch inhibitor and Hh pathway inhibitor GANT61 in combination with the natural anticancer drug (-)-Gossypol (Gos) reduces the expression of stemness markers and prevents sphere formation and recovery [116]. The gene expression profile of tumor samples from GBM patients indicates that Notch2 transcripts are positively correlated with the transcription of genes that control anti-apoptotic processes, stem, and astrocytic glioma cell fate [117].

Sun Z et al. found that the neurosphere formation, proliferation, invasiveness, and tumor formation abilities of normal GBM cells were significantly enhanced after treatment with GSC exosomes. Concurrently, Notch1 signaling was activated in GSC; Notch1 signaling and expression of stemness-associated proteins were increased in normal GBM cells treated with GSC exosomes and in the resulting tumor tissues [118]. The expression of Netrin-1 promotes activation of Notch signaling and alters the phenotype of non-invasive GBM cells, causing them to transition into a diffuse invasive state and increasing the expression of GSC markers [119]. In GBM, Macrophage Colony-Stimulating Factor (MCSF) is upregulated and recruits macrophages into the tumor microenvironment to support tumor growth [120]. 5-FU treatment-induced reduction of cancer stem cell proportion in U87-MCSF cells is reversed by upregulation of Notch-1 inducing EMT in U87-MCSF cells [121].

Self-renewal and stem cell differentiation mediated by the Notch signaling pathway are also manifestations of glioma cell stemness [122]. FAM129A is a positive regulator of Notch signaling through binding to NICD1. Targeting FAM129A inhibits GSC self-renewal [123]. Simultaneous inhibition of Notch and Wnt/β-catenin pathway in GSCs with elevated ASCL1 expression promotes significant neuronal differentiation while suppressing the clonogenic potential [124]. Knockdown of the *IGFBP2* gene significantly affects the cell cycle, Notch pathway, neural stem cell differentiation, and the expression of differentiation genes in neural stem cells [125]. ZNF117 regulates GSC differentiation to oligodendrocytes by the Notch signaling pathway [126]. Deletion of ADAM17 in U87 GSCs inhibits Hes1 and Hes5 while activating Notch1 expression, thereby inducing self-renewal and inhibiting differentiation in U87 GSCs [127]. Delta/Notch-like epidermal growth factor-related receptor (DNER) is a specific gene product induced by HDAC inhibition. Its noteworthy influence encompasses the inhibition of glioblastoma (GBM)-derived neurosphere growth and the induction of differentiation in both in vitro and in vivo settings [128]. The treatment of GSC with γ-secretase inhibitor (GSI) induces a phenotypic shift to non-tumorigenic cells [129].

Additionally, TMZ can also mediate the Notch signaling pathway to regulate GBM stemness. TMZ promotes the expression of the EGR1 transcription factor, which binds to the MMP14 promoter to promote MMP14 expression and nuclear translocation and leads to the extracellular release of DLL4, which in turn stimulates Notch3 cleavage and nuclear translocation and induces sphere-forming capacity and stemness in GSCs [130]. Overexpression of LNX1 leads to Notch1 signaling activation, and GSCs increase after TMZ treatment [131].

## 4. Status of Targeted Notch Therapy

### 4.1. Clinical Trials Targeting Notch in Cancer

Elucidation of the details of the Notch signaling pathway has facilitated the development of inhibitors and activators targeting different levels of the pathway, ranging from small-molecule inhibitors to antibody-based antagonists and agonists. To date, most translational efforts have been based on the repurposing of γ-secretase inhibitors (GSIs), which were originally developed not as Notch-targeting agents but as inhibitors of the hydrolytic cleavage of the β-amyloid precursor protein (β-APP), which generates amyloidogenic peptides and plays a central role in the pathogenesis of Alzheimer‘s disease [132,133].

The frequent identification of gain-of-function mutations in NOTCH1 in T-cell acute lymphoblastic leukemia/lymphoma (T-ALL) prompted the rapid development of the first clinical trial of Merck‘s GSI drug MK-0752 in patients with relapsed/refractory T-ALL [134]. As with other early GSI trials, this study was open to all patients, regardless of their T-ALL Notch mutation status. In addition, the daily dosing schedule used in this trial resulted in severe diarrhea, which justified the dose restriction and was likely due to the known Notch toxicity of GSIs, which is the enhancement of intestinal cuprocyte differentiation at the expense of intestinal epithelial cell differentiation [135,136]. Subsequent GSI trials by Merck, Roche (RO4929097), Pfizer (PF-03084014), and Bristol-Myers Squibb (BMS-906024) used a staged dosing regimen that avoided intestinal toxicity and were largely safe and well tolerated [137,138,139], but again did not use tumor “Notch status” as a criterion for enrollment. The major disappointment of these trials was the relatively low level of evidence of anti-tumor activity, although there were a few exceptions. Two patients with relapsed/refractory T-ALL achieved complete hematologic remission; notably, both had strong gain-of-function mutations involving the NOTCH1 negative regulatory region (NRR) [138,140]. There is also evidence that a small proportion of patients with adenomyomas respond to GSIs [141,142]; these are aggressive fibroblastic tumors that are not known to have gain-of-function mutations in the Notch gene. The mechanism by which adenomyomas respond to GSIs is unknown and may be mediated by the cleavage effect of GSIs on γ-secretase substrates (non-Notch receptors).

A gamma secretase inhibitor, CB-103, is in clinical development [143]. As a direct inhibitor of Notch-induced transcription of downstream target genes, it has a faster onset of action, particularly in tumors harboring Notch receptors with deletions in the PEST structural domain, but may not achieve the same depth of inhibition because NICD production is not affected. In principle, these compounds could also target tumors harboring specific Notch gene abnormalities, such as rearrangements involving NOTCH2 in breast cancer, which are resistant to targeted therapy with GSIs [144]. Interestingly, preclinical data suggest that CB-103 is unlikely to cause intestinal toxicity [143]; whether this reflects differences in mechanism of action compared to GSIs or other properties of this molecule remains to be determined. In addition, a Phase I/II clinical trial (NCT03422679) of CB-103 in patients with locally advanced or metastatic solid tumors and hematologic malignancies is ongoing. In this trial, CB-103 is administered orally for 28 days per treatment cycle, with dose escalation in Part A (Phase I) of the trial, followed by a dose increase in Part B (Phase IIA).

Based on the central role of Notch in angiogenesis and the hypothesized role of Notch in cancer stem cells, Genentech [145], Regeneron [146], and OncoMed developed antibody antagonists to DLL4. DLL4-blocking antibodies resulted in excessive, dysregulated angiogenesis and the formation of abnormal blood vessels that were ineffective at transporting nutrients, thereby inhibiting tumor growth in preclinical models [147]. A Phase I trial of Regeneron‘s anti-DLL4 antibody Enoticumab in patients with solid tumors showed some evidence of disease stabilization in a subset of patients but also resulted in cardiotoxicity and hypertension [146], which may reflect the role of DLL4/Notch in the vascular endothelium. OncoMed‘s anti-DLL4 antibody study with demcizumab also produced hypertension and showed no evidence of efficacy in Phase II studies in patients with pancreatic cancer and non-small-cell lung cancer. OncoMed also conducted a Phase II study with tarextumab, an antibody that blocks the activation of both NOTCH2 and NOTCH3, and again, no antitumor activity was observed in small-cell lung cancer [148].

The widespread and specific expression of the Notch inhibitory ligand DLL3 on SCLC tumor cells makes it an ideal therapeutic target [149]. Currently, DLL3-targeted therapy occupies a dominant position compared to other therapies targeting Notch signaling. Rovalpituzumab tesirine (Rova-T) is an experimental antibody-drug conjugate (ADC) that specifically targets DLL3 in the Notch signaling pathway. The mechanism of action of Rova-T is that the antibody (rovalpituzumab) binds specifically to the DLL3 protein on the surface of cancer cells. Once bound, the ADC is internalized by the cancer cell, and the cytotoxic drug (tesirine) is released inside the cell. This drug then induces DNA damage, leading to the death of the cancer cell [150]. Good response and survival rates were observed in early studies (NCT01901653 [151] and NCT02674568 [152]), but the phase III trial of Rova-T (TAHOE, NCT03061812) was stopped due to inferior overall survival (OS) of 6.3 months in the Rova-T group compared to 8.6 months in the topotecan group and a higher incidence of adverse events (AEs) [150]. Another phase III study (MERU, NCT03033511) showed that the median OS for patients taking Rova-T was also not superior to the placebo group (8.5 months vs. 9.8 months) [153], leading to the termination of further Rova-T studies.

### 4.2. Clinical Trials Targeting Notch in Glioblastoma

Although there have been many reports of clinical trials targeting different levels of inhibitors in the Notch signaling pathway, only four clinical trials have been reported on a γ-secretase inhibitor (RO4929097) in glioblastoma.

In a Phase 0/I trial (NCT01119599), 21 patients with newly diagnosed glioblastoma or anaplastic astrocytoma were treated with RO4929097 in combination with temozolomide and radiotherapy. The results showed that patients tolerated the treatment well, and no dose-limiting toxicities were observed. The IHC of the tumor after treatment showed a significant reduction in tumor cell and vascular proliferation and Notch intracellular structural domain (NICD) expression. Patient-specific organotypic tumor explant cultures showed a significant reduction in the CD133+ CIS population after treatment [154].

In a Phase II clinical trial (NCT01122901), patients with recurrent GBM were treated with RO4929097 in two groups: patients in Group A had unresectable disease and were treated with the drug according to a standard Phase II design, and patients in Group B had resectable disease and were treated with the drug before and after surgical resection. A total of 47 patients were treated, of which 7 had resected tumors with a 6-month progression-free survival rate (PFS6) of 4%, and 1 of the 7 patient samples inhibited neurosphere formation. The results showed that RO4929097 was inactive in patients with recurrent GBM and had minimal inhibition of neurosphere formation in fresh tissue samples [155].

The Adult Brain Tumor Consortium conducted a Phase I clinical trial (NCT01189240) in patients with recurrent malignant glioma to evaluate and determine the safety and maximum tolerated dose of RO4929097 in combination with bevacizumab. A total of 13 subjects were enrolled in this study. One grade III toxicity and one grade II toxicity were observed in the three subjects treated with the highest dose of RO4929097. Of the 12 evaluable subjects, two patients had radiographic responses; one subject had a CR and the other had a PR. The median overall survival was 10.9 months, and the median progression-free survival was 3.7 months. The results showed that the combination of RO4929097 with bevacizumab was well tolerated [156].

To determine the safety and maximum tolerated dose of RO4909297 administered at two dose levels and the pharmacokinetics, intratumoral drug concentrations, target modulation, and evidence of any therapeutic effect of RO4909297 in malignant glioma tumor tissue in patients with recurrent MG, many of whom are treated with dexamethasone, a moderate CYP3A4 inducer. The investigators conducted a phase I clinical trial (NCT01269411). However, there are no results or literature reports on this clinical trial.

### 4.3. Targeting Notch in GBM

Notch inhibitors have significant potential in the treatment of glioblastoma (Table 1). There are three main categories: The γ-secretase inhibitor (GSI), the most widely used, functions by preventing the release of active NCID from the receptor by inhibiting the γ-secretase complex. α-secretase inhibitor (ASI), less commonly used, works by inhibiting members of the ADAM family. In addition, due to the nature of the tumor, long-term medication leads to GSI/ASI resistance, and researchers have explored a number of other molecular inhibitors that target the Notch signaling pathway.

#### 4.3.1. γ-Secretase Inhibitors in GBM

DAPT is the most commonly used GSI. Inhibiting Notch signaling with DAPT can alter VEGF receptor expression, thereby suppressing GBM growth [157]. GBM neurospheres with elevated Notch activity show increased sensitivity to DAPT and exhibit a more pronounced phenotype with DAPT therapy [158,159]. In GBM, targeting Notch 1 with DAPT reduces NF-κB (p65) expression, induces apoptosis, and inhibits cell proliferation [91]. In primary human glioblastoma cells, DAPT can partially rescue the aberrant Notch signaling caused by *lipin 1* knockdown [160]. The combination of STAT and DAPT therapies led to a significant increase in apoptosis in the cells being treated, resulting in a severe impairment of cell proliferation, migration, and invasion [161]. In comparison with monotherapy, the combination treatment of DAPT with Iressa (an EGFR inhibitor) reduces the expression and secretion of VEGF, which significantly enhances the elimination of endothelial cell sprouting generated by GBM [162]. Different from DAPT, LLNle kills GBM tumor-initiating cells by inhibiting proteasome activity. Furthermore, LLNle and L-685,458 can also induce proteolytic stress and mitotic arrest by blocking NICD generation [163]. L-685,458 is able to significantly reduce CXCR4 mRNA in endothelial cells stimulated with rhDLL4 [164]. Under the combined treatment of Resveratrol (RSV) and RO4929097, CDK4 inhibits the population of glioblastoma stem cells with a more invasive phenotype, also triggers apoptosis by pathways blocking the autophagic flux [83,165]. The treatment was well tolerated, and the combination of RO4929097, temozolomide, and RT had a specific decrease in the CD133+ CIS population [154].

The blockade of Notch signaling, which is targeted by GSI-I, can enhance the radiosensitivity of glioblastoma cell lines, inhibit xenograft and tumor neurosphere growth, and lead to the depletion of CD133-positive glioblastoma cells [166]. Research suggests that MRK003 has significant therapeutic potential for CD44-high and CD133-low GICs and that it is mediated in part by the Akt pathway [167]. MRK003 induces protective autophagy in glioma neurospheres, and combination therapy with chloroquine reverses this phenomenon [168]. Blocking Notch with MRK003 has been shown to have a broad impact on the metabolism of brain tumor cells, including glycolysis, glutamine, and choline metabolism and dopamine degradation [169]. The NOTCH blockers MRK003 and GSI-XVII reduced the growth and clonogenicity of neurospheres in vitro. NOTCH blockade was followed by a reduction in the specific CSC markers CD133, NESTIN, BMI1, and OLIG2 [167,170]. In GBM, GSI-X inhibited the cell growth of some c-CSC but had minimal effect on their apoptosis, cell cycle distribution and cell invasion [77].

#### 4.3.2. α-Secretase Inhibitors in GBM

Compared to GSI, there has been very limited research on ASIs, which target the cleavage of the NECD surface proteins ADAM10 and ADAM17 [171]. Desiree H. et al. suggested that ASI (INCB3619) treatment is similar to GSI and proposed several new targets for ASI and GSI in GBM stem cells (GSCs), which are crucial for tumor growth. These novel targets include LIF and CHI3L1/YKL-40. The expression of CHI3L1/YKL-40 serves as an adverse prognostic indicator in many cancers and inflammatory diseases [65].

**Table 1:** Inhibitors for the treatment of glioblastoma via Notch signaling.

Class	Molecules	Testing Index	Biological Effects
γ-Secretase inhibitor (GSI)	DAPT(GSI-IX) [158]	Notch1; DLL1; VEGF	Improving sensitivity to radiotherapy; inducing cell differentiation.
	LLNle [163]	Notch1; Notch2; Hey2; Myc; Akt	Inducing cell apoptosis.
	L-685,458 [164]	Hes1	Inhibiting neurosphere formation.
	RO4929097 [154]	Notch2; Notch4	Inhibiting neurosphere formation; reducing drug resistance.
	MRK003 [167]	Akt	Reducing drug resistance; inducing cell apoptosis.
	GSI-XVII [170]		Inducing cell apoptosis.
	GSI-I [166]		Improving sensitivity to radiotherapy.
	GSI-X [77]	Notch1	Inducing cell apoptosis; suppressing cell invasion.
α-Secretase inhibitor (ASI)	INCB3619 [65]	Notch1; p21; p53	Inhibiting cell proliferation.
Other molecular inhibitor	Arsenic trioxide (ATO) [75]	Notch1; Hes1	Improving sensitivity to radiotherapy.
	AG1478 [172]	Notch1; EGFR	Inducing cell apoptosis.
	Retinoic acid [173]	Hes2; Hey1; Hey2	Inducing neural differentiation; inhibiting cell proliferation.
	N-acetylcysteine [174]	Notch2; Hes1; Hey2	Inhibiting cell proliferation; suppressing cell invasion and migration.
	dnMAML [175]	Hes1; Hey-L	Inhibiting cell proliferation; inducing cell apoptosis.
	Honokiol [176]	Notch3; Hes1	Promoting the pharmacodynamics of TMZ.
	Bacoside A [108]	Notch1; Hes1	Inducing cell apoptosis.
	Hypocretin-1 [109]	Notch1	Inducing cell apoptosis.
	Resveratrol [110]	Notch1; p53	Inducing cell apoptosis.

#### 4.3.3. Other Molecule Inhibitors

Besides ASI and GSI, several other molecular inhibitors have been found to block the Notch signaling pathway in glioblastoma.

Arsenic trioxide (ATO) is an inorganic compound that was approved by the FDA in 2000 for the treatment of refractory relapsed acute promyelocytic leukemia (APL) due to its potent anti-acute promyelocytic leukemia (APL)-derived stem cell growth [177]. Zhang et al. found that arsenic trioxide (ATO) can deplete cancer stem-like cells (CSLCs) and inhibit the repopulation of GBM-derived neurospheres. This effect was associated with the downregulation of the Notch pathway, which plays a central role in the maintenance of CSLCs in GBM [75]. Meanwhile, ATO disrupts GSCs and inhibits the growth of orthotopic xenografts derived from GSCs, which promotes the degradation of promyelocytic leukemia (PML) protein, resulting in increased apoptosis in GSCs. This suggests that ATO may effectively suppress tumor growth by targeting key molecular components within GSCs [178].

AG1478 is a molecularly targeted inhibitor of the epidermal growth factor receptor (EGFR). It is widely used in cancer therapy and cancer cell research. In a study by Carlo Cenciarelli et al., it was found that a single application of the Notch inhibitor in GBM significantly inhibited the growth of c-cancer MSCs compared to p-cancer MSCs but had little effect on the induction of apoptosis, cell cycle distribution, and cell invasion assays. In contrast, single application of anti-epidermal growth factor receptor (AG1478) therapy induced cell cycle arrest, sometimes associated with apoptosis, and reduced cell invasiveness of CSCs. Moreover, the combined application of GSI-X and AG1478 induced apoptosis in nuclear mesenchymal stem cells [172].

Research suggests that retinoic acid (RA) inhibits the activation of Notch signaling, thereby downregulating the expression of Hes and Hey family members and inducing growth arrest and differentiation of GBM neurospheres in vitro and in vivo [173]. N-acetylcysteine (NAC) inhibits glioblastoma cell proliferation, migration, and invasion and promotes Notch2 degradation, possibly through an Itch-dependent lysosomal pathway, while decreasing mRNA and protein levels of its downstream target genes, Hes1 and Hey1, thereby inducing apoptosis [174]. 

Dominant negative MAML (dnMAML) inhibits Notch signaling activity, binds to elastin-like polypeptide (ELP), and targets tumors. Further modification with the cell-penetrating peptide SynB1 enhances cellular uptake and blood–brain barrier penetration. Under high-temperature conditions, the pharmacological action of dnMAML can be enhanced. SynB1-ELP1-dnMAML inhibits glioblastoma cell growth by inducing cell cycle arrest and apoptosis, and it inhibits Notch pathway targets such as Hes-1 and Hey-L [175].

### 4.4. Notch and Glioblsatoma Resistance

Glioblastoma is a common primary brain tumor of the central nervous system in adults. Throughout the last decade, advancements in treatment have been gradual, and the prognosis continues to be exceedingly unfavorable [179]. Temozolomide (TMZ) is a prodrug for the treatment of GBM. It effectively traverses the blood–brain barrier and is metabolized in the body to an active metabolite. This metabolite methylates DNA, thereby inhibiting the replication and transcription of tumor cells [180]. However, a significant obstacle for TMZ is the emergence of drug resistance in tumor cells. Long-term use of TMZ can also be associated with cumulative toxicities. Therefore, it is urgent to focus on elucidating the molecular mechanisms underlying drug resistance and developing strategies to treat it.

Recent studies have demonstrated the molecular mechanisms of GBM resistance from the perspective of Notch signaling and TMZ. EFEMP1 is an activator of Notch signaling that is overexpressed in TMZ-resistant GBM cells and enhances GBM cells that are resistant to TMZ [181]. KDELC2 is able to downregulate the expression of Notch factors, including Hes-1, pofut1, Notch receptors 1-3, and KDELC1. The knockdown of *KDELC2* in combination with TMZ therapy achieves optimal therapeutic efficacy by suppressing Methylguanine-DNA methyltransferase (MGMT) expression in GBM [182]. LncRNA *LINC00021* is significantly upregulated in GBM, especially in tissues and cells resistant to TMZ, and is closely associated with TMZ resistance and poor prognosis [183]. Mechanistically, transcription factor E2F1 activated the expression of *LINC00021*, which regulates glioblastoma resistance to TMZ via the Notch pathway. It also epigenetically represses the expression of p21 by recruiting EZH2 [183]. Deletion of PLK2 in GBM leads to activation of the Notch pathway by negatively regulating Hes1 transcription and promoting Notch1 degradation. Low PLK2 expression predicts a poor prognosis and resistance to TMZ in GBM patients [184]. In the co-treatment of glioblastoma cells with human astrocytes using TMZ and Bay 11-7082 (a NF-κB inhibitor), the expression levels of GFAP, waveform protein, Notch1, and survivin were significantly reduced. However, the astrocyte presence elevated the survival rate and resistance of glioblastoma cells to the combined drug treatment [185]. LNX1 functions as an E3 ubiquitin ligase that acts upon Numb. Treatment with TMZ increases the binding of LNX1 to Numb and the ubiquitination of LNX1, thereby regulating Notch signaling through the LNX1–Numb–Notch1 axis, which further influences therapeutic resistance in GBM [131].

Honokiol is a bioactive compound primarily derived from the bark of the magnolia tree, and it has been used in traditional oriental medicine for numerous years, especially in China and Japan. Honokiol exhibits a variety of pharmacological properties, encompassing anti-inflammatory, antioxidant, anxiolytic, and anti-tumor effects [176]. The activity of MGMT is particularly significant in cancer treatment. When cancer cells exhibit high levels of MGMT, they can repair the damage induced by alkylating chemotherapy agents effectively, such as TMZ, leading to drug resistance. I-Chun Lai et al. discovered that the combined use of the MGMT inhibitor O6-BG and Honokiol can reduce the expression of TMZ-induced Notch3 and Hes1 mRNAs, potentially reversing the resistance of glioblastoma stem-like cells (GBM SP cells) to TMZ [186].

Radiation therapy (RT) is another treatment option for GBM. However, patients who receive RT for long periods of time may develop tumor resistance, and the effectiveness of RT is limited and varies from person to person [187,188]. The combination of GSI, RT, and TMZ can inhibit cell proliferation, neurosphere formation, and survival in both primary and established glioma cell lines. It also reduces the GSCs markers expression such as CD133, SOX2, and nestin [189]. RT-induced changes in the tumor microenvironment are also one of the challenges in treating GBM [190]. G9a promotes filtration of IFN-γ and CD4+ and CD8+ T lymphocytes in GSCs and binds to the Notch suppressor Fbxw7, which inhibits gene transcription via H3K9me2 from the *Fbxw7* promoter [191].

## 5. Summary and Prospects

The Notch signaling pathway serves as a critical intercellular communication mechanism in multicellular organisms, activated by various stimuli and involving a complex interplay of signaling pathways. Recent years have witnessed significant advancements in our understanding of the role of Notch signaling in various aspects of GBM biology, including growth, proliferation, invasion, migration, therapeutic strategies, apoptosis, drug resistance, and stemness. Particularly, numerous studies consistently implicate the PI3-AKT/NF-κB/β-catenin/Notch axis in GBM progression. The Notch pathway primarily involves nuclear transcription of NCID and subsequent activation of downstream target genes, such as the Hes/Hey family. Hence, the Notch signaling pathway plays a crucial role in regulating GBM growth and proliferation, particularly in the self-renewal and differentiation capacities of GSCs.

However, while many studies have shown that certain genes can influence the growth, migration, and invasive capabilities of GBM cells by activating the intracellular Notch signaling pathway, there is insufficient evidence to demonstrate direct binding of these genes to factors in the Notch pathway or their direct participation in the pathway. Therefore, future research should focus on deeper exploration of the expression of downstream target genes and the molecular regulatory networks of the Notch signaling pathway in GBM rather than relying solely on phenomenological experiments.

Understanding the molecular mechanisms of carcinogenesis and signaling pathways holds great promise for clinical practice, with the Notch pathway offering potential targets for therapeutic intervention. In recent years, therapeutic strategies targeting the Notch pathway have focused on the development of antibody drugs, antibody-drug conjugates (ADCs), and small-molecule γ-secretase inhibitors (GSIs). Despite some positive results from early clinical trials targeting specific Notch receptors and ligands, challenges such as individual differences in therapeutic efficacy, dose-limiting toxicity, and drug resistance remain significant hurdles.

In GBM, GSIs are the most common and effective inhibitors, preventing the activation of the γ-secretase complex and thereby inhibiting the formation of active Notch forms. Additionally, α-secretase inhibitors and other molecular inhibitors are being explored. While targeting Notch with single therapies appears promising, recent studies have focused on inducing apoptosis in GBM cells with natural compounds or overcoming GBM cell resistance with the chemotherapy drug TMZ. Therefore, future studies should aim to deepen our understanding of the specific mechanisms of action of the Notch signaling pathway in different cancers and address the side effects and resistance issues associated with current therapeutic approaches. Combining cutting-edge research on the Notch signaling pathway with small-molecule compounds could pave the way for the design of targeted drugs and provide more options for clinical GBM therapy. In conclusion, the lack of clinical trials emphasizes the necessity of conducting such studies.
